# Pentatonic sequences and monaural beats to facilitate relaxation: an EEG study

**DOI:** 10.3389/fpsyg.2024.1369485

**Published:** 2024-04-15

**Authors:** Marco Costa, Chiara Visentin, Miranda Occhionero, Lorenzo Tonetti, Nicola Prodi, Vincenzo Natale

**Affiliations:** ^1^Department of Psychology “Renzo Canestrari”, University of Bologna, Bologna, Italy; ^2^Department of Engineering, University of Ferrara, Ferrara, Italy

**Keywords:** pentatonic sequences, beats, silence, relaxation, EEG

## Abstract

**Introduction:**

In two studies we investigated if specific acoustic stimulations could be more effective to induce a relaxation response in comparison to silence. Acoustic stimulations included monaural beats and musical sequences based on a pentatonic scale.

**Methods:**

In the first study, 47 participants evaluated monaural beats and pentatonic sequences presented through loudspeakers and varying along three frequencies (0.2, 2, 4 Hz). In the second study, 31 participants relaxed with their eyes closed for 10 min during a passive listening of monaural beats and a pentatonic sequence presented through loudspeakers. A silence condition was introduced as control. All auditory stimuli were designed with a temporal modulation of 0.2 Hz. Concomitant EEG was recorded with a 64-channel system and spectral analysis was performed on delta, theta, alpha, beta, and gamma oscillations to test if each of the three auditory stimulations had a significant effect on EEG spectral power in comparison to silence.

**Results:**

In the first study, pentatonic sequences were evaluated as more pleasant and more relaxing than monaural beats. Pleasantness and relaxation were inversely related to frequency. Visual imagery and emotion induction had higher frequency and were rated with a more positive valence in pentatonic sequences than in monaural beats. In the second study monaural beats in comparison to silence strongly decreased beta and gamma oscillations in the first three minutes and strongly increased theta oscillations in the last three minutes. Pentatonic sequences increased delta, theta, and alpha oscillations in the last three minutes while decreasing beta, and gamma oscillations for the whole auditory stimulation.

**Discussion:**

The results show that auditory signals with a very low temporal modulation (0.2 Hz) could be more effective than silence in inducing a relaxation response. Although 0.2 Hz monaural beats were effective in inducing a relaxation response, they tended to be perceived as unpleasant. Pentatonic sequences could be considered as a better alternative to promote relaxation by auditory stimulation.

## Introduction

Can specific auditory stimulations be more effective than silence in inducing a relaxation response? Relaxation is a specific psychological state of low tension, with a reduction of arousal, a shift for selective to diffuse attention, and a prevalence of parasympathetic autonomous activity (Friedman et al., 1989; Kokoszka, 1992). Relaxation is often achieved through a reduction of sensorial stimulation and a withdrawal from external inputs, but people often use specific low-intensity, repetitive stimuli to gain a better state of relaxation. For example, a relaxation response can be induced through the repetition of words, the use of repetitive music, and meditation often includes the repetition of gestures or words (Hoffman et al., 1982; Chang et al., 2010). In this paper, we focused on the auditory domain comparing the relaxing potential of silence with the relaxing potential of two specific auditory stimulations, one based on monaural beats and the other consisting of musical sequences based on the pentatonic scale.

Relaxing potential was assessed by both self-assessment and measures of the spectral distribution of the EEG signal. The most consistent finding regarding the EEG spectral correlates of relaxation is an increase in theta activity (e.g., Kasamatsu and Hirai, 1966; Warrenburg et al., 1980; Jacobs and Friedman, 2004). The effect of relaxation on alpha activity is more controversial. Although the majority of studies have reported an increased alpha activity (e.g., Lagopoulos et al., 2009; Mikicin and Kowalczyk, 2015), others have reported decreased alpha (Stigsby et al., 1981; Jacobs and Lubar, 1989). Several researchers have underlined the similarities between the relaxation response, stage 1 sleep, and the hypnagogic state (Elson et al., 1977; Fenwick et al., 1977).

Much of the previous research in this field has not directly compared silence with a specific acoustic stimulation but has often investigated the effect of specific sounds or musical excerpts on relaxation. The categories of auditory stimuli that have received specific attention in previous studies are musical excerpts, standardized broad-band sounds (e.g., white, pink noise), natural sounds (e.g., rain, wind, water flow), and beats (monaural or binaural). In our study, we focus on very basic musical sequences and beats. We did not include standardized sounds because previous studies are rather controversial in their effects on relaxation and sleep onset (Cordi, 2021). We did not include natural sounds because rain, wind, and water flow sounds can be considered variations of broad-band sounds.

Music is certainly one of the most frequent aid that people choose for achieving a relaxing state. Clinical and laboratory-based studies revealed that listening to music can decrease sympathetic activity (Bartlett, 1996; Hodges, 2010). Music therapists have consistently used music as a stimulus to induce a relaxation response (e.g., Madson and Silverman, 2010; Warth et al., 2016). The effects of relaxing music on EEG spectral distribution have been investigated by Paszkiel et al. (2020) who have compared the impact of rap, relaxing music, ASMR-triggering sounds, and silence on stress level. Relaxing music and ASMR sounds increased the magnitude of alpha waves more than silence. Rap music was the least efficient way to calm down. Kabuto et al. (1993) found a positive association between the relaxation effects of pleasant music and the total theta power and the alpha power in the occipital region.

Although music can be very effective in inducing calmness, relaxation, and sleep, the effect is strongly related to music preference (Jiang et al., 2013, 2016), and music preference is highly subjective. Therefore, it would be impossible to generalize and propose specific musical excerpts that would be valid for a general audience. Furthermore, tones in music are presented with different probabilities, following harmonic hierarchies, and being some tones more frequently presented than others (for example, in a transitory modulation, very infrequent tones can be temporarily introduced). This uneven distribution can elicit an involuntary capture of attention by rare sounds that would inhibit relaxation or sleepiness (Andrés et al., 2006).

Beats have drawn the attention of researchers in the last years with encouraging results. Auditory beats are signals with an amplitude modulation, generated by mixing two sine waves with a fixed amplitude and a slight difference in frequency (between 2 and 30 Hz, Perrott and Nelson, 1969). The signal is perceived as a beat, with an amplitude modulation at a frequency equal to the tones frequency difference. Beats can be either monaural or binaural. In the case of monaural beats, the two sine waves are mixed in a single audio stream and then presented to both ears simultaneously. Monaural beats are physical beats, directly processed at the cochlea level. In binaural beats, the two tones are presented separately to the left and right ears. This phenomenon is particularly interesting since the summation of tones is operated by the brain (Oster, 1973). Although produced in two different ways, the perception of beats is present whenever the production method (binaural, monaural), so the results obtained with binaural beats can be largely extended to monaural beats and vice versa.

Studies that have applied continuous EEG recording with the application of beats have shown changes particularly in the gamma (Pastor et al., 2002; Schwarz and Taylor, 2005) and alpha bands (Vernon et al., 2014). At the core of the effects of beats is that they can elicit an entrainment effect in the electrocortical activity of the brain, a phenomenon referred to as frequency following response (FFR) which arises from the brain stem (Hink et al., 1980). Significant electrocortical entrainment due to beats has been reported for delta (Pratt et al., 2010), theta (Brady and Stevens, 2000; Karino et al., 2006; Pratt et al., 2010), and gamma (Schwarz and Taylor, 2005).

Gao et al. (2014) tested the effects of 5-min beats presentation in the delta, theta, alpha, and beta band on the relative power spectrum of the EEG signal. They observed a relative power increase in theta and alpha bands and a decrease in the beta band during delta and alpha beats stimulations. Relative power decreased in the beta band during theta stimulation, while relative power decreased in theta band during beta binaural beats.

Although beats seem rather effective in modulating EEG spectral distribution, their repetitive, monotonous, and unnatural sound could make people feel uncomfortable (Recuero et al., 2013; Engelbregt et al., 2021). Their repetitivity can cause anxiety and depression (Watkins, 2008). To resolve this problem, beats have been combined with other sounds that are perceived as more pleasant (Wiwatwongwana et al., 2016; Gantt et al., 2017), as, for example, ASMR sounds (Lee et al., 2019).

Most of the previous studies that have investigated the effects of beats on relaxation and sleep onset have used oscillations in the delta and theta range. Auditory stimulations with oscillations in the lower end of delta (or sub-delta) waves (i.e., < 0.5 Hz) have never been investigated. It is therefore of great interest to establish if auditory oscillations that are outside of the delta range could entrain the EEG and could have an effect on EEG spectral power in comparison to auditory stimulations in the delta/theta bands.

Besides varying acoustical stimulations for tempo/speed we also compared monaural beats to pentatonic sequences. These sequences were designed as a succession of notes belonging to a pentatonic scale. This scale consists of five notes per octave, in contrast to the more common heptatonic scale. Most important this scale lacks semitone intervals, therefore the tonal tension toward the tonic is much lower, creating a sensation of vagueness, indetermination, and fluctuation. Specifically, we used a C minor pentatonic scale (C3, Eb3, F3, G3, Bb3, C4) because minor music tends to be perceived as less arousing, more contemplative, and sadder (Parncutt, 2014). Furthermore, the scale was chosen in the middle-low register (C3–C4), avoiding high-pitch tones that are mostly associated with alarm, tension, and arousal (McAdams et al., 2017; Belluscio et al., 2023). In fact, high-pitch tones are frequently used to alarm and capture attention (e.g., Straughn et al., 2009; Belluscio et al., 2023). To keep the timbre as simple as possible pure tones were used.

We conducted two studies. The first study assessed with self-report scales (relaxation, pleasantness, valence of induced imagery and emotion) acoustical stimulations that varied systematically for tempo (notes/oscillations at 4, 2, 0.2 Hz) and method (beats versus pentatonic sequences). The hypothesis was that pentatonic sequences would be evaluated as more pleasant and inducing more pleasant imagery and emotions than beats.

In a second study, monaural beats and pentatonic sequences were directly compared to a control “silence” condition assessing EEG spectral power in the delta, theta, alpha, beta, and gamma EEG bands. While the first study was mainly aimed at the choice of the frequency of oscillation/note succession that was mostly associated with relaxation, the second study was mainly aimed to assess if monaural beats and pentatonic sequences were more effective in increasing theta and/or delta EEG power than silence and if EEG spectral perturbations induced by beats were significantly different from EEG spectral perturbations induced by pentatonic sequences.

The final aim of both studies was the development and testing of auditory stimulations that could be embedded in apps or electronic devices that could facilitate people in relaxing, lowering high arousal states, and improving sleep onset.

## Study I

### Methods

#### Participants

Participants were 47 university students (*M*_age_ = 24.1, *SD* = 3.5, range: 20–33), 16 females and 31 males. The students were mainly enrolled in engineering courses at the University of Ferrara. A preliminary interview excluded the presence of hearing loss or hearing diseases. None of the participants was professional in the musical domain or had received formal music training for more than 10 years. The study was approved by the Bioethics Committee of the University of Bologna (Protocol # 0250471 10/17/2022) and the signing of an informed consent was requested from all participants.

#### Stimuli and material

Sound stimuli to be assessed by participants were divided into two main categories: monaural beats and pentatonic sequences. For each category three frequencies were presented (0.2, 2, 4 Hz).

**Figure 1:**
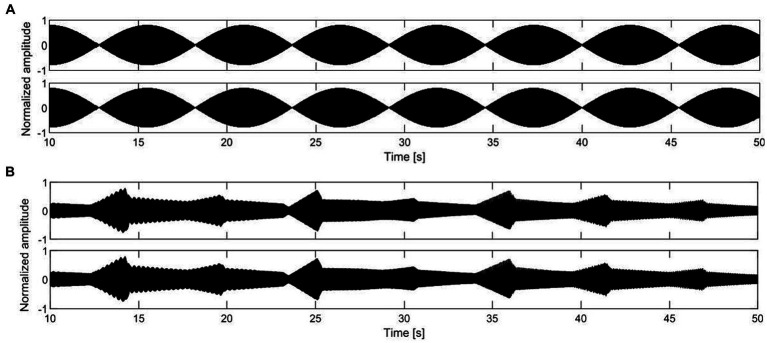
Energy-time graphs of a selection of the auditory stimuli used in the first study, with a temporal modulation of 0.2 Hz: **(A)** monaural beats, **(B)** pentatonic sequence.

Monaural beats were designed by mixing a base sine wave at 130.8 Hz with an additional sine wave differing 0.2, 2, and 4 Hz (131, 132.8, and 134.8 Hz, respectively). Figure 1A shows the envelope in the case of 0.2 Hz. Two simultaneous pure tones with frequencies within the same critical band are perceived as a stimulus with amplitude modulation at the frequency of the difference between the two inputs (Chaieb et al., 2015).

Pentatonic sequences consisted of a melodic succession of notes belonging to the C minor pentatonic scale (C3, Eb3, F3, G3, Bb3, C4). The octave range was C3 (131 Hz) and C4 (262 Hz). Notes had a pure tone timbre. The envelope included a 40% rise time with a parabolic profile, and a 60% decay time with a linear profile. To smooth the transition between notes the linear decay did not reach 0 amplitude but 30% of the peak amplitude (Figure 1B). Single note duration was 5 s in the 0.2 Hz condition, 0.5 s in the 2 Hz condition, and 0.25 s in the 4 Hz condition. The repetition of the same note was avoided, as well as the dissonant seventh minor interval C3 – Bb3.

All acoustic stimuli were reproduced using a binaural rendering system installed in a silent room at the Department of Engineering of the University of Ferrara (Italy). The loudspeaker system surrounds the listener and consisted of four pairs of loudspeakers lying on the same plane and with different angular spans (three pairs in the frontal arch and one in the rear). The sound stimuli were presented at a level of 37.5 dB(A), measured with reference to the ears of a head-and-torso simulator (B&K4100) placed at the listener’s position. The participant used a touchscreen to input the responses.

Acoustic stimuli were assessed on the following scales: (a) pleasantness (unpleasant-pleasant); (b) relaxation (relaxing-arousing); (c1) the ability to evoke visual imagery (yes/no); (c2) in case of “yes” response to the previous question, the valence of visual imagery (negative–positive); (d1) the ability to evoke emotions (yes/no); (d2) in case of “yes” response to the previous question, the valence of the evoked emotions (negative-pleasant). Visual analog scales ranging from 0 to 100 were used to acquire the responses. Audio presentation and data acquisition was managed through an in-house developed LabVIEW routine.

As a potential covariate we also assessed sensitivity to noise with the Italian version (Senese et al., 2012) of the *Weinstein’s Noise Sensitivity Scale* (Weinstein, 1978). The WNSS was administered in its short version, that consists of five items (items 21, 20, 19, 18, 8 of the long version). Responses were on a six-point Likert scale ranging from totally disagree to totally agree. The mean score of the five items indicates the sensitivity to noise, with higher scores reflecting a higher sensitivity.

#### Procedure

After the presentation of the study, and the signing of the informed consent the participant was interviewed for the assessment of auditory deficits. After completion of the *Weinstein’s Noise Sensitivity Scale*, the participant was moved to the sound-attenuated room and instructed about the response device. Each sound was presented for 60 s. During the presentation the participant was invited to close her/his eyes, trying to relax. After 30 s, with the sound stimulus still played back, the participant could start evaluating the sound on the six scales previously described. This 30-s time delay in the response was functional to allow the participant to initially focus exclusively on the stimulus and then provide a judgment based not only on memory but also on simultaneous listening. Once the evaluation was completed for all the scales, the next stimulus was automatically played. The total duration of the study was approximately 30 min. The order of presentation of the stimuli was counterbalanced across participants, and each stimulus was presented and evaluated only once.

#### Data analysis

Statistical analyses were conducted with the R software (v.4.3.1). For each assessment scale, two-way repeated-measure ANOVAs were performed to evaluate the effect of frequency over the two acoustic stimuli. Independent variables were frequency (three levels: 0.2, 2, 4 Hz) and type of stimulus (two levels: monaural beats, pentatonic sequence). The two-way interaction was always included in the models. Covariate was the score in the *Weinstein’s Noise Sensitivity Scale*. Dependent variable was the rating for the specific scale under examination. Greenhouse–Geisser correction was applied to reduce sphericity issues.

### Results

#### Pleasantness

**Figure 2:**
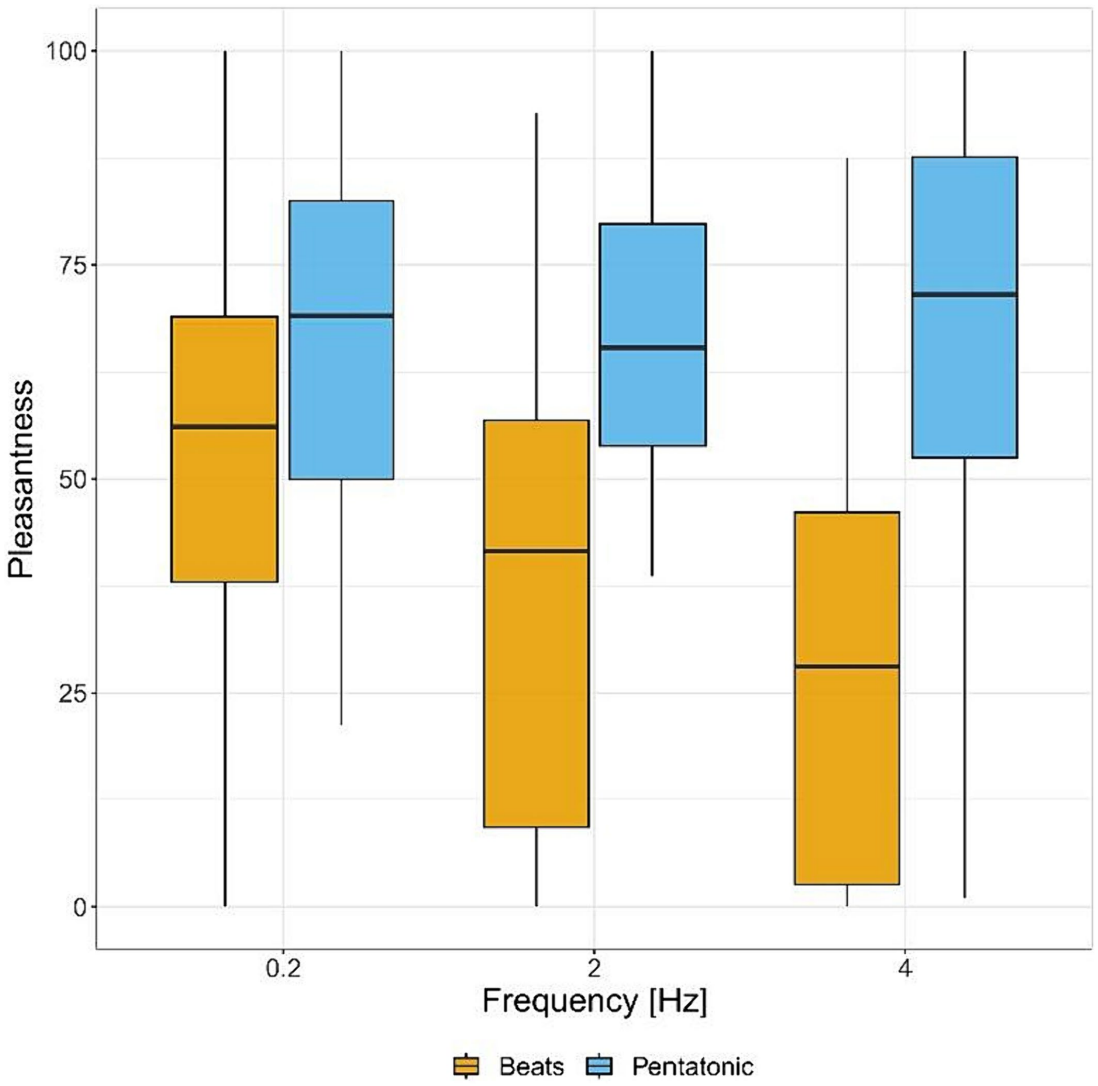
Pleasantness ratings (from unpleasant to pleasant) for each sound stimulus by frequency.

Figure 2 and Table 1 show pleasantness ratings for the two stimuli, by frequency. In the pleasantness assessment there was a statistically significant interaction between frequency and type of stimulus: *F*(2.00, 88.00) = 11.62, *p* < 0.001, η^2^ = 0.06. Consequently, an analysis of simple main effects for frequency level was performed, with statistical significance receiving a Bonferroni adjustment. Pairwise comparisons showed that beats were rated significantly more unpleasant than pentatonic sequence at all frequencies (0.2 Hz, *p* = 0.006, *d* = 0.53, 2 Hz, *p* < 0.001, *d* = 1.34, 4 Hz, *p* < 0.001, *d* = 1.78). Pairwise comparisons were also performed between the different frequencies organized by stimulus type. For beats, there was a significant decrease of pleasantness with increase of frequency (0.2 Hz > 2 Hz, *p* = 0.009, *d* = 0.65, 2 Hz > 4 Hz, *p* = 0.02, *d* = 0.40). For pentatonic sequences the difference between frequencies was not statistically significant.

**Table 1:** Mean (standard deviation) for the ratings in the beats and pentatonic sequence condition, by frequency.

		Beats	
	0.2 Hz	2 Hz	4 Hz
Pleasantness	53.5 (25.9)	36.1 (27.8)	26.1 (22.5)
Relaxation	67.5 (26.5)	35.7 (27.3)	35.0 (30.3)
Imagery valence	56.7 (28.1)	42.5 (24.6)	41.6 (19.3)
Emotion valence	59.0 (27.9)	38.8 (26.4)	30.0 (16.4)
		Pentatonic sequence	
Pleasantness	66.3 (22.7)	67.2 (19.2)	68.4 (24.5)
Relaxation	80.1 (20.1)	57.0 (26.8)	41.3 (32.8)
Imagery valence	67.2 (26.6)	68.8 (17.3)	77.4 (17.6)
Emotion valence	66.4 (25.8)	65.6 (24.5)	69.6 (26.6)

#### Relaxation

**Figure 3:**
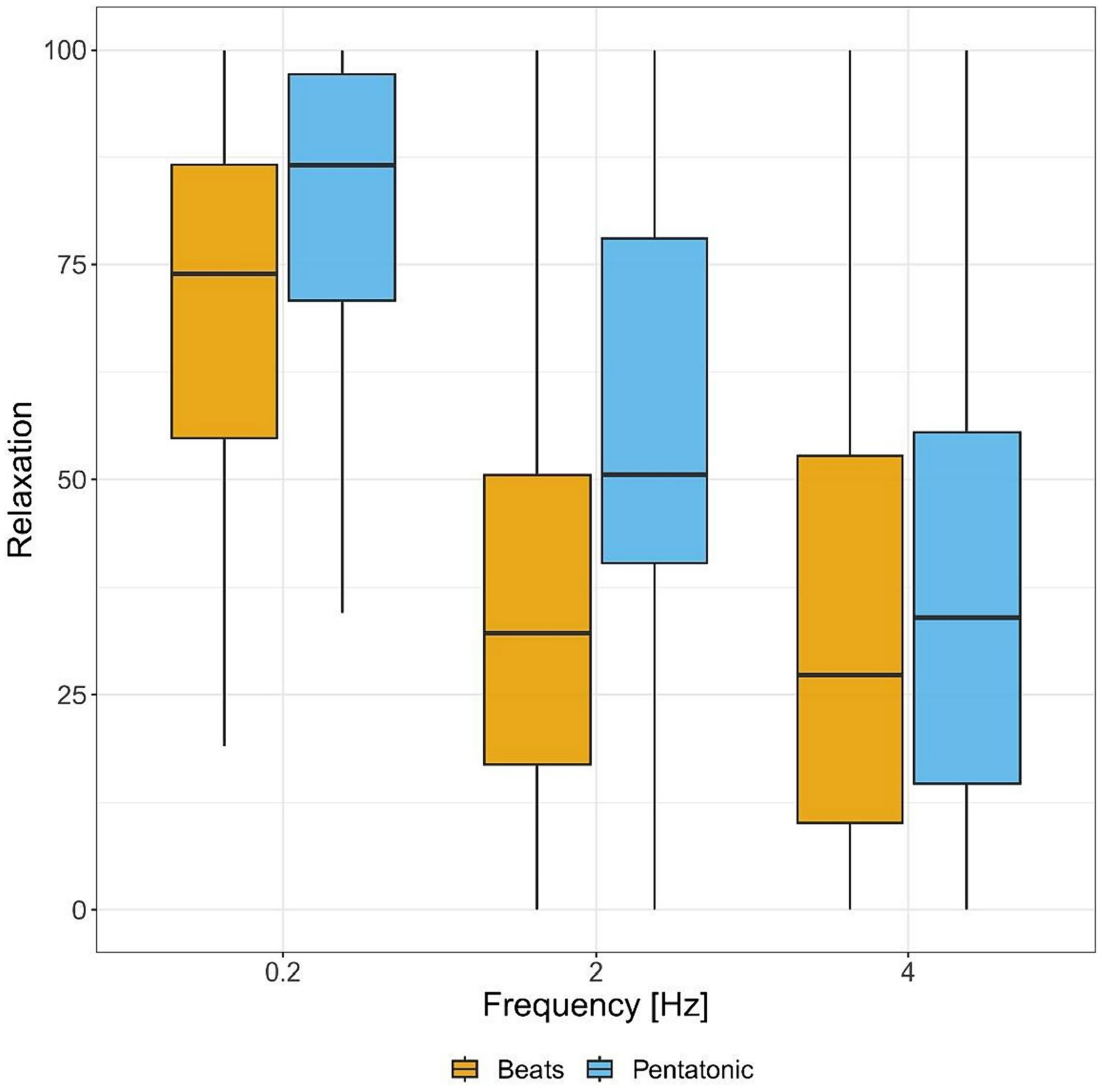
Relaxation ratings (from relaxing to arousing) for each sound stimulus by frequency.

Figure 3 and Table 1 show relaxation ratings for the two stimuli, by frequency. In the relaxation assessment there was a statistically significant main effect of the type of stimulus, indicating that beats were rated as less relaxing than pentatonic sequence: *F*(1.00, 44.00) = 12.24, *p* < 0.001, η^2^ = 0.06. The main effect of frequency was also statistically significant: *F*(1.82, 79.98) = 68.45, *p* < 0.001, η^2^ = 0.25. Specifically, the relaxation ratings decreased with the increase of the sound stimulus frequency (*p* < 0.001 for all comparisons, 0.2 Hz > 2 Hz: *d* = 1.03, 2 Hz > 4 Hz: *d* = 0.27).

#### Visual imagery

The induction of visual imagery in the beats condition was reported by 29.8% at 0.2 Hz, 44.7% at 2 Hz, and 51.1% at 4 Hz. In the pentatonic sequence condition, induction of visual imagery was reported by 27.7% at 0.2 Hz, 51.1% at 2 Hz, and 57.4% at 4 Hz. Proportion comparisons using binomial distribution showed that the two types of stimuli induce the same rate of visual imagery, while stimuli at 0.2 Hz induce a significantly lower rate of visual imagery than the other two frequencies.

**Figure 4:**
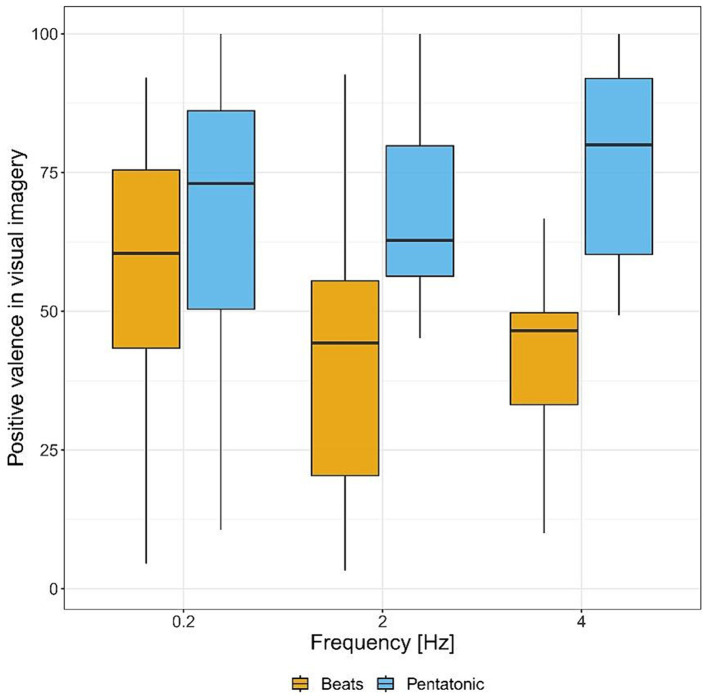
Positive valence of visual imagery ratings (from negative to positive) for each sound stimulus by frequency.

Figure 4 and Table 1 show imagery valence ratings for the two acoustic stimuli, by frequency. Imagery valence differed significantly as a function of the type of stimulus: *F*(1.00, 104.97) = 36.85, *p* < 0.001, η^2^ = 0.06. Specifically, it was significantly more positive in the pentatonic sequence than in the beats condition.

#### Emotions

The induction of emotions in the beats condition was reported by 48.9% at 0.2 Hz, 53.2% at 2 Hz, and 53.2% at 4 Hz. In the pentatonic sequence condition, emotion-induction was reported by 57.4% at 0.2 Hz, 55.3% at 2 Hz, and 70.2% at 4 Hz. Proportion comparisons using binomial distribution showed that pentatonic sequence induced a significantly higher rate of emotions than beats, while the effect of frequency was non-significant.

**Figure 5:**
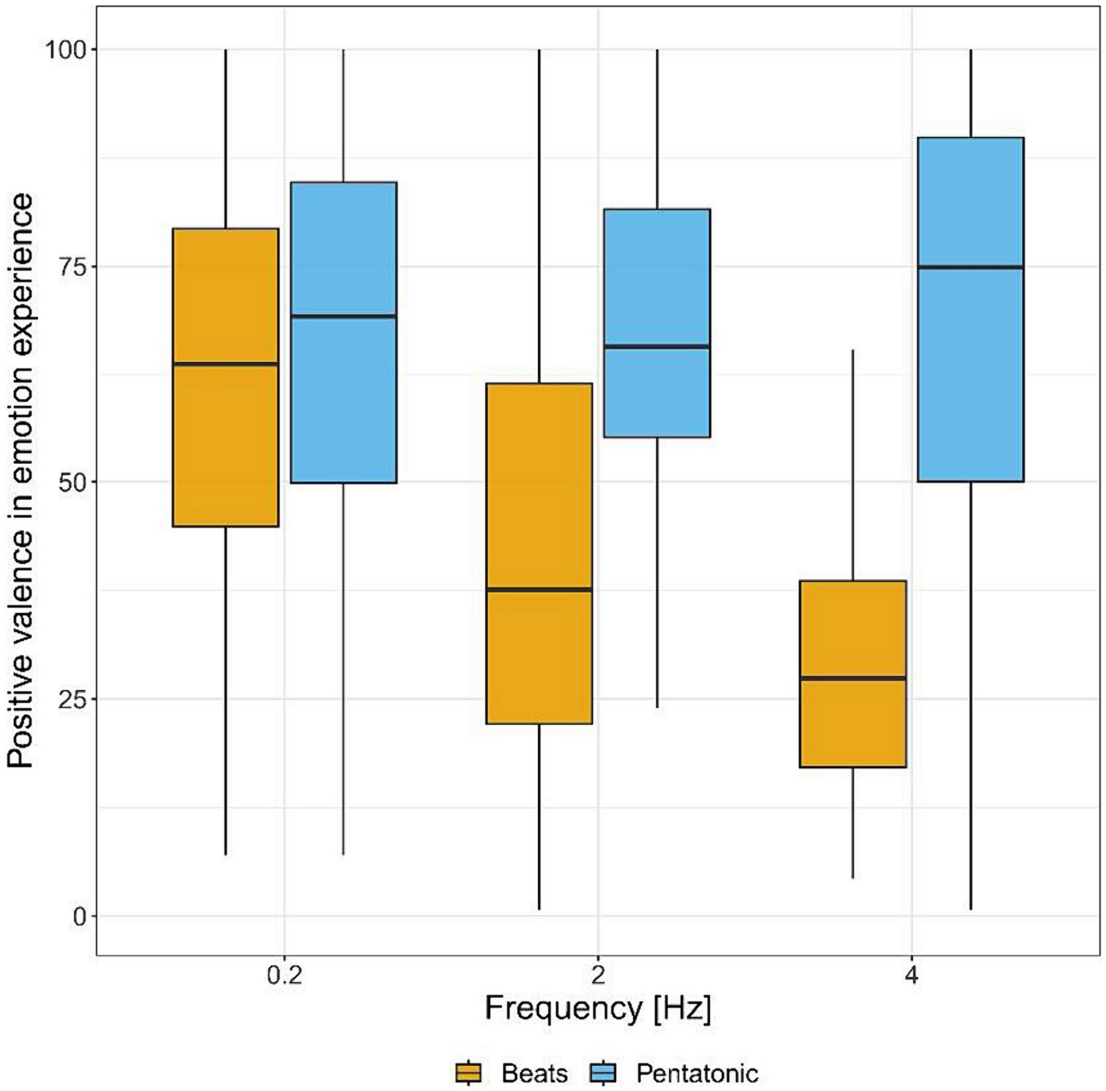
Positive valence in emotion experience (from negative to positive) for each sound stimulus by frequency.

Figure 5 and Table 1 show valence ratings in emotion experience for the two acoustic stimuli, by frequency. There was a statistically significant interaction between frequency and type of stimulus: *F*(2.00, 2.00) = 23.86, *p* < 0.04, η^2^ = 0.18. Consequently, an analysis of simple main effects for frequency level was performed, with statistical significance receiving a Bonferroni adjustment. Pairwise comparisons showed that beats induced more negative emotions than pentatonic sequence at 2 and 4 Hz (*p* < 0.001 for both comparisons, 2 Hz: *d* = 1.05, 4 Hz: *d* = 1.79), while there was no statistically significant difference at 0.2 Hz (*p* = 0.61). Pairwise comparisons were also performed between the different frequencies organized by stimulus type. For beats, emotion experience was significantly more positive at 0.2 Hz than 2 Hz (*p* = 0.02, *d* = 0.74) and 4 Hz (*p* < 0.001, *d* = 1.27) while the comparison between 2 and 4 Hz was not significant (*p* = 0.70). For pentatonic sequence the difference between frequencies was not statistically significant (*p*s > 0.38).

##### Weinstein’s noise sensitivity scale

Mean Weinstein’s Noise Sensitivity Scale score was 3.77 (*SD* = 1.01). The score was positively correlated with age (*r* = 0.18, *p* = 0.003), and negatively correlated with pleasantness ratings (*r* = −0.13, *p* = 0.036) and imagery valence (*r* = −0.14, *p* = 0.015). As covariate in the previous ANOVAs, it was never significant.

### Discussion study I

The results of the first study showed a marked preference for pentatonic sequences in comparison to beats, for all the three frequencies considered in the study. As highlighted also in previous studies (Recuero et al., 2013; Engelbregt et al., 2021), beats tend to be perceived as very monotonous, repetitive. For this reason, their use in applied context such as apps or devices seems problematic. On the contrary pentatonic sequences were evaluated as more pleasant, probably due to their higher musicality and complexity.

When the sound evoked visual imagery, the valence of the imagery was more positive for pentatonic sequences than beats. Furthermore, emotions were experienced more frequently and had a more positive valence when induced by pentatonic sequences than beats.

An inverse linear relation was found between self-evaluated relaxation and frequency of the auditory stimulation. Specifically, 0.2 Hz was evaluated as the more relaxing frequency. This frequency is equivalent to a metronome setting of 12 bpm, a tempo that is usually considered too slow in music. For this reason, it can be considered a supernormal stimulus that exaggerates the relation between slow tempo and relaxing music (Hernandez-Ruiz et al., 2020).

Since the results of this study pointed to 0.2 Hz as the acoustic oscillation that was best associated with relaxation, positive imagery and positive emotions, we decided to focus on this oscillation in the next study in which we examined a 10-min exposure to 0.2 Hz monaural beats and pentatonic sequences with EEG recording and EEG spectral power computation associated to delta, theta, alpha, beta, and gamma bands. A 10-min exposure to silence was introduced as a control condition. The aim of the second study was to assess if monaural beats and/or pentatonic sequences were more powerful than silence in modifying the EEG power spectrum increasing the alpha/theta/delta bands that are mostly associated with relaxation, hypnagogic state, and drowsiness in comparison to beta and gamma bands.

## Study II

### Methods

#### Participants

Participants were 31 (*M*_Age_ = 26.48, *SD* = 7.90), 22 females and 9 males, mainly university students from psychology courses at the University of Bologna. A preliminary interview excluded hearing loss or hearing diseases. None of the participants was professional in the musical domain or had received formal music training for more than 10 years. The study was approved by the Bioethics Committee of the University of Bologna (Protocol # 0250471 10/17/2022) and the signing of an informed consent was requested to all participants.

#### Stimuli and material

Stimuli consisted of two auditory sequences having in common a basic oscillation at 0.2 Hz, and a control silence condition. Auditory sequences included monaural beats and pentatonic sequences. The sound design of monaural beats and pentatonic sequences mirrored exactly the one used in Study I. Supplementary material includes 1-min extract of the two acoustical stimuli used in Study II.

All acoustic stimuli were presented with a sound level of 34 dB(A) assessed with a Delta Ohm HD2010 sound level meter. This level was chosen sufficiently low to facilitate relaxation and sleepiness. Left and right speakers were positioned at 130 cm from left and right ear, respectively, forming an angle of 80° with the participant positioned in the vertex of the triangle. Sensitivity to noise was assessed as covariate with the *Short Weinstein Sensitivity Scale* (Senese et al., 2012).

#### Procedure

After the presentation of the study, the exclusion of hearing problems with an interview, and the signing of the informed consent the participant was requested to complete the *Short Weinstein Noise Sensitivity Scale*. The professional expertise or education in the musical domain (yes/no) and the level of vigilance on a visual-analog scale (scoring from 0 to 100) were also recorded.

The participant was prepared for the EEG recording applying a BIOSEMI headcap with 64 active EEG sensors spatially distributed according to the 10–20 system, two sensors applied to the left and right mastoid, and applying the CMS and DRL sensors for the BIOSEMI EEG acquisition system. Vertical eye movements were recorded with a sensor applied below the left eye in bipolar association with the Fp1 sensor. Horizontal eye movements were recorded with two sensors positioned in the outer canthi of the left and right eye. The participant was seated in an armchair and instructed to relax with eyes closed for an interval of 14 min. The sampling rate was 256 Hz. The order of conditions (silence, beats, pentatonic sequence) was randomized between participants.

Each listening condition followed this procedure: 2 min of silence as an adaptation time, 10 min acoustic stimulus presentation (or silence in the silence condition), 2 min silence. This sequence mirrored the procedure used in Lee et al. (2019). A ten-minute interval separated the different conditions. During this interval the participants was request to evaluate the condition on the following scales: (a) pleasantness-unpleasantness of the condition (visual-analog scale); (b) the ability of the condition to evoke visual imagery (yes/no), and, in case of “yes” response, if the visual images were negative or positive (visual-analog scale); (c) the ability of the condition to evoke emotions (yes/no); (d) in case of “yes” response to the previous question, the valence of the emotion (visual-analog scale ranging from “Positive” to “Negative”). The participant was also asked to assess the ability of the condition to facilitate relaxation (visual-analog scale). The total duration of the study was approximately 90 min.

#### Data analysis

Self-report data were analyzed with repeated-measure ANOVAs with condition as independent variable. Eta-squared was used as an effect size index in ANOVAs results. Cohen’s *d* was used for computing effect size in planned-comparisons. The scores of the *Short Weinstein Noise Sensitivity Scale* were inserted as covariate. Greenhouse–Geisser correction was applied to reduce sphericity problems. EEG preprocessing, and statistical analysis were performed using EEGLAB (Delorme and Makeig, 2004), Fieldtrip (Oostenveld et al., 2011), MATLAB with Statistics and Machine Learning toolbox, and SPSS. Data preprocessing followed a standard pipeline, which included re-referencing to linked mastoids, downsampling to 256 Hz, low-pass filtering (100 Hz), and high-pass filtering (0.05 Hz) for the reduction of DC offset. Two steps were performed to minimize the artifacts caused by eye blinks, muscle activity, eye movements, and heartbeat activity. First, artifacts due to massive interference from sources outside the scalp were detected using a moving window peak-to-peak algorithm. This excluded all trials in which a peak-to-peak excursion greater than 150 μV was detected in an EEG channel. Moving window width was set to 200 ms, and window step was set to 100 ms. Second, EEG data were decomposed applying an independent component analysis on EEG channels only. The components were classified according to the ICLabel algorithm (Pion-Tonachini et al., 2019). The components were discarded according to these thresholds: eye-movement (*p* > 0.90), channel noise (*p* > 0.90), line-noise (*p* > 0.90), heart (*p* > 0.90), and muscle activity (*p* > 0.60).

Spectral decomposition was performed using the Welch method (Welch, 1967) with Hanning window. The EEG signal was split into 10 s windows with a 50% overlap. The overlapping segments were Hanning windowed, and the periodogram was computed for each segment applying a discrete Fourier transform. The coefficients were squared obtaining spectral magnitude and the individual periodograms were then averaged, thus reducing the noise and variance of individual spectral power measurement. Spectral analysis was performed separately on the first 3 min and on the last 3 min, to better investigate the dynamic evolution of EEG oscillations during the auditory stimulation (or silence). The silence condition was considered as a control condition.

To illustrate the variations in EEG oscillations induced by each auditory stimulation, we computed dB of the ratio between power spectral density for the auditory condition and power spectral density for the silence condition, according to the Formula 1 where *n* was the number of samples entered in the analysis.


(1) $$\text{dB}=10\;\text{log}\;\frac{PSD\;\text{Conditio}n^{2}/n}{PSD\;\text{Silenc}e^{2}/n}$$

Spectral analysis was performed considering the following bands: delta (0.5–4 Hz), theta (4.5–7.5 Hz), alfa (8–12 Hz), beta (12.5–30 Hz), and gamma (31–45 Hz). Entrainment was tested by assessing spectral power at 0.2 Hz. Statistical analyses for the EEG were performed by adopting a non-parametric approach with Montecarlo permutations (Zhu, 2005; Maris and Oostenveld, 2007). The number of permutations for each analysis was set to 10,000. The multiple comparison problem was controlled with the cluster method proposed by Maris and Oostenveld (2007).

### Results

#### Pleasantness

**Figure 6:**
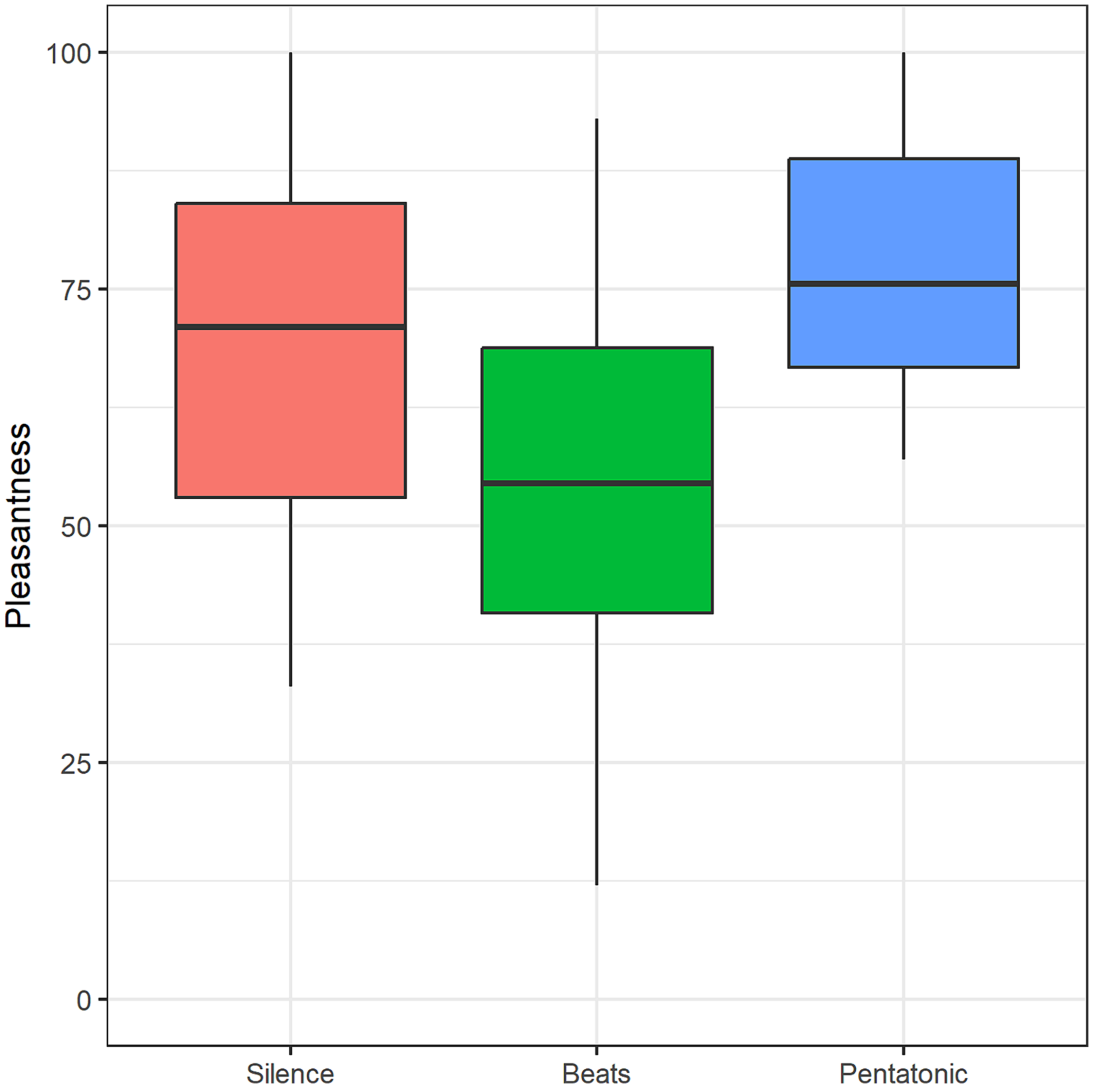
Pleasantness mean rating as a function of condition.

Pleasantness assessment differed significantly between conditions: *F*(2, 41) = 5.94, *p* = 0.005, η^2^ = 0.14. Figure 6 and Table 2 show mean pleasantness ratings for the three conditions. Pairwise comparisons showed that beats were rated significantly more unpleasant than silence (*p* = 0.02, *d* = 0.84) and pentatonic sequence (*p* = 0.008, *d* = 0.79).

**Table 2:** Mean (standard deviation) for the ratings in the silence, beats, and pentatonic sequence condition.

	Silence	Beats	Pentatonic sequence
Pleasantness	74.50 (16.74)	58.07 (21.77)	74.60 (19.55)
Imagery valence	54.15 (28.88)	54.00 (13.87)	71.62 (18.69)
Emotion valence	56.56 (27.38)	49.25 (21.37)	73.38 (16.48)
Relaxation	78.10 (17.52)	58.77 (25.42)	70.47 (19.03)

#### Imagery

The induction of visual imagery was reported by 43.33% in the silence condition, 61.3% in the beats condition, and 67.7% in the pentatonic condition. Proportion comparisons using binomial distribution showed that the silence condition induced a significantly lower rate of visual imagery than all the auditory conditions.

**Figure 7:**
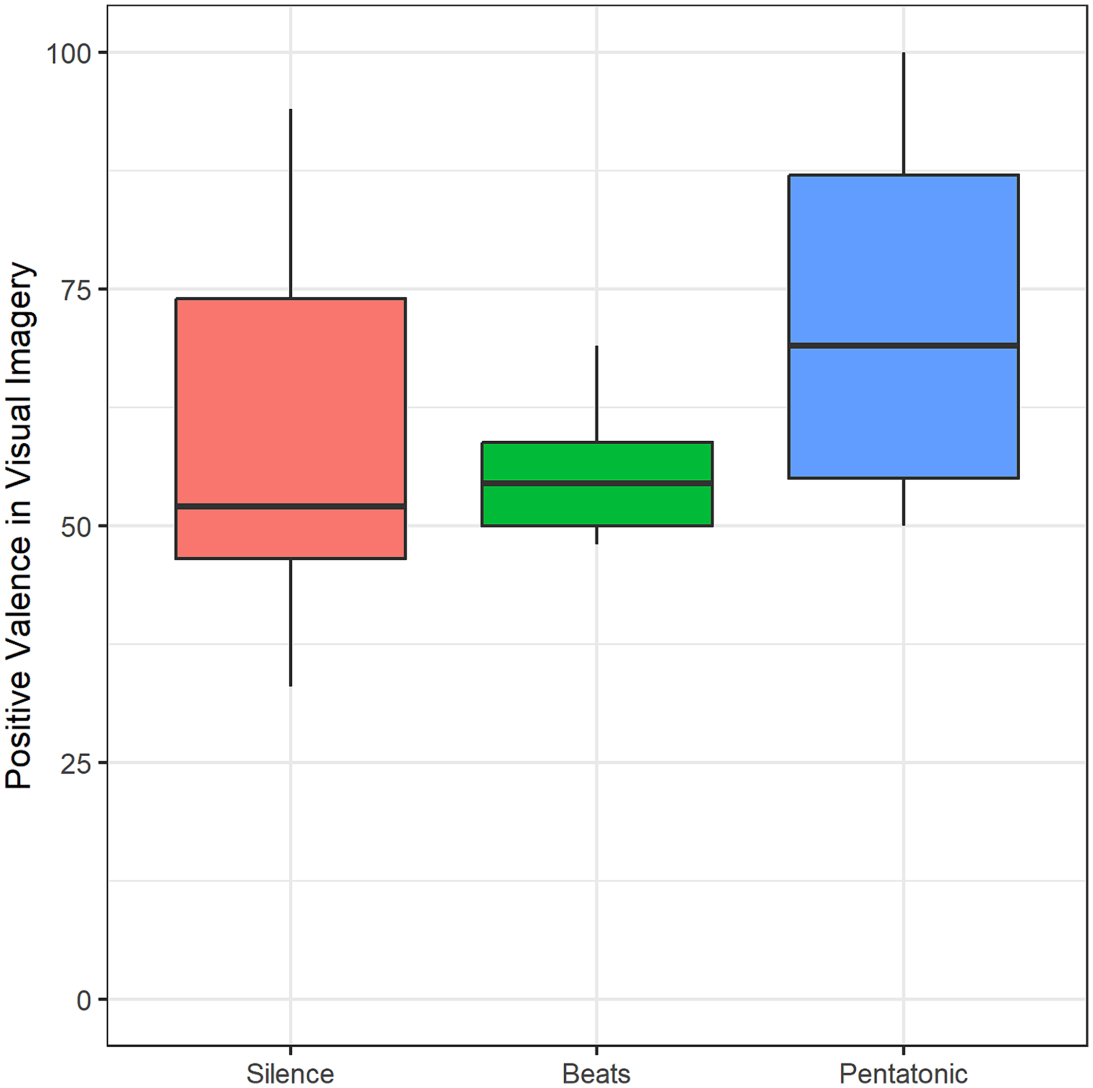
Positive valence of visual imagery mean rating as a function of condition.

Imagery valence differed significantly as a function of Condition: *F*(2, 23) = 4.57, *p* = 0.02, η^2^ = 0.17. Imagery valence was significantly more positive in the pentatonic sequences than in the silence condition (*p* = 0.05, *d* = 0.71) and in the beats condition (*p* = 0.04, *d* = 1.07). Figure 7 and Table 2 show mean imagery valence ratings for the three conditions.

#### Emotions

The frequency of emotion-induction in the three conditions was: 50% in the silence condition, 61.3% in the beats condition, and 77.4% in the pentatonic condition.

**Figure 8:**
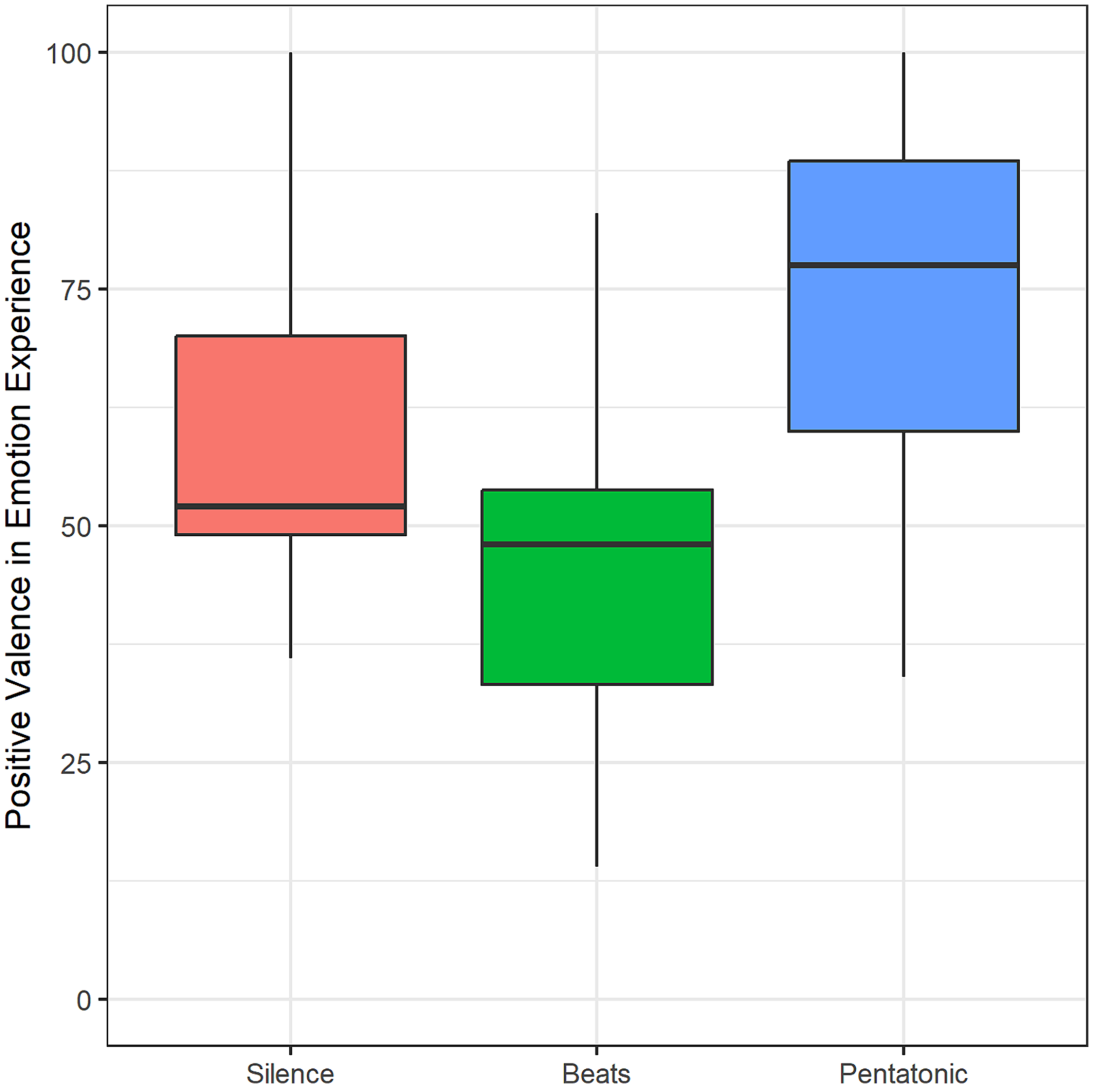
Positive valence in emotion experience as a function of condition.

Condition significantly influenced also the valence of the experienced emotion: *F*(1.63, 21.21) = 5.05, *p* = 0.02, η^2^ = 0.28. Specifically, emotions induced by beats had a more negative valence than emotions induced by silence (*p* = 0.01, *d* = 0.29) and pentatonic sequence (*p* = 0.01, *d* = 1.26). Figure 8 and Table 2 show mean valence ratings for the three conditions.

#### Relaxation

**Figure 9:**
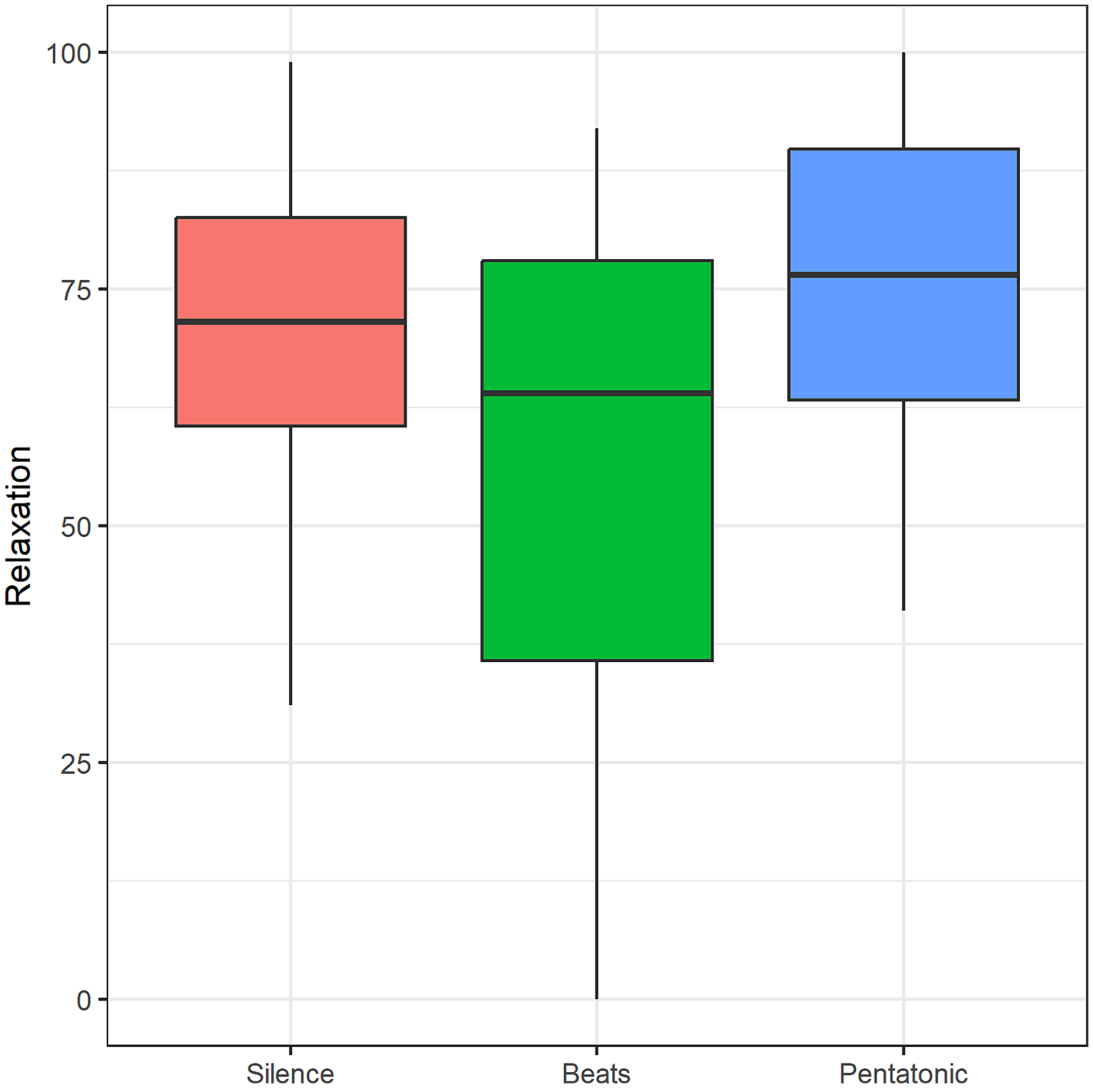
Relaxation mean ratings as a function of condition.

Relaxation was significantly affected by Condition: *F*(2, 42) = 4.43, *p* = 0.01, η^2^ = 0.12. Relaxation in the pentatonic condition was rated significantly higher than in the beats condition (*p* = 0.01, *d* = 0.52). Figure 9 and Table 2 show mean relaxation ratings for the three conditions.

#### Short Weinstein Noise Sensitivity Scale

The mean Short Weinstein Noise Sensitivity Scale score was 4.09 (*SD* = 0.99) on a 1–6 range. Short Weinstein Noise Sensitivity Scale score was inversely significantly correlated with age (*r* = −0.36, *p* = 0.04), and with imagery valence in the silence condition (*r* = −0.56, *p* = 0.01). As covariate in the previous ANOVAs, it was never significant.

#### Vigilance level

The mean self-assessed vigilance level was 60.23 (*SD* = 23.16). It was directly correlated with pleasantness ratings in the Beats condition (*r* = 0.38, *p* = 0.03). As a covariate in the previous ANOVAs, it was never significant.

#### EEG spectral analysis

##### Entrainment 0.2 Hz

No significant differences were found in spectral power for 0.2 Hz between the two auditory conditions and silence.

##### Beats/silence—topography of EEG power spectrum

**Figure 10:**
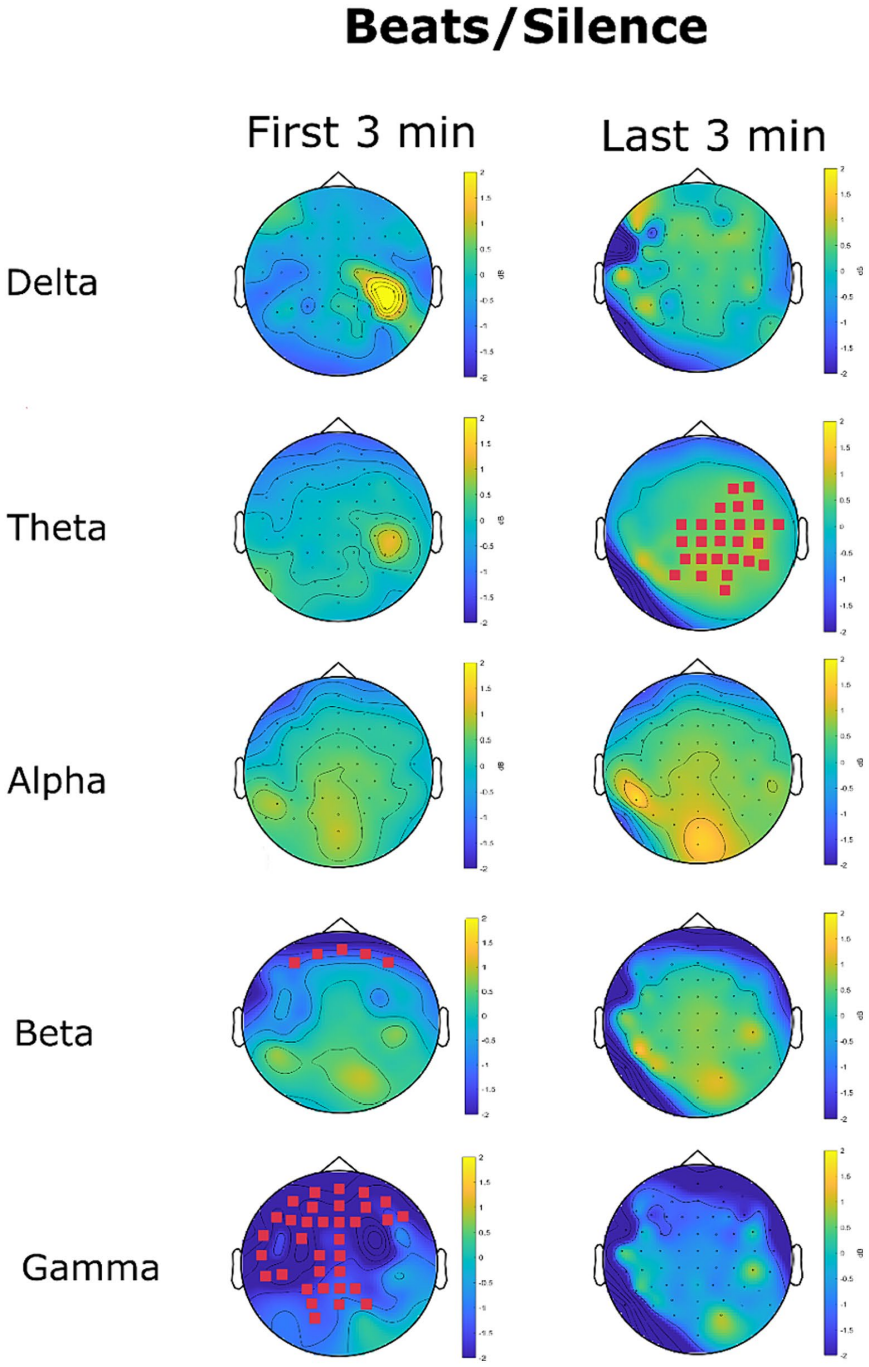
EEG power spectrum change (dB) in the beats condition with reference to the silence condition. Magenta channel marks highlight the channel with a significant difference in power spectrum between the two conditions.

Figure 10 shows the change in EEG oscillations in the beats condition in comparison to the silence condition. Statistical analysis at the channel level clearly showed an early effect in which beats reduced high-frequency oscillations, particularly in the beta (12.5-30 Hz) and gamma (31–45 Hz) range. A late effect, manifest in the last 3 min, was an increase of slow-wave oscillations particularly in the theta range.

No significant differences were found for delta oscillations. A significant cluster involving 26 channels for theta increase oscillations for beats was recorded in the last 3 min. The mean power spectrum was 0.28 μV^2^/Hz (*SD* = 0.09) in the silence condition and 0.33 μV^2^/Hz (*SD* = 0.11) in the beats condition (*p* = 0.007). This effect related to theta oscillations exhibited a marked right hemisphere lateralization (69.23% of significant channels in the right hemisphere).

No significant differences were found for alpha oscillations. Beta oscillations were significantly lowered in the beats condition in the first 3 min (*M_Silence_ =* 0.13 μV^2^/Hz *SD_Silence_ =* 0.03, *M_Beats_ =* 0.10 μV^2^/Hz *SD_Silence_ =* 0.03, *p* = 0.04, *d* = 1). The significant cluster included five channels in the frontal region: Fp1, AF7, Fpz, Fp2, and AF8, as shown in Figure 10. No significant differences were found for beta oscillations in the last 3 min.

Gamma oscillations were strongly reduced in the beats condition in the first three minutes. This reduction interested 34 channels covering quite all the frontal-central-temporal sensors, with an accentuated left lateralization for central and temporal sensors. The gamma power spectrum decreased from 0.04 μV^2^/Hz (*SD* = 0.02) in the silence condition to a mean of 0.02 μV^2^/Hz in the beats condition (*p* = 0.004, *d* = 1). The decrease in gamma oscillations was not significant considering the last three minutes (*p* = 0.17).

##### Pentatonic/silence—topography of EEG power spectrum

**Figure 11:**
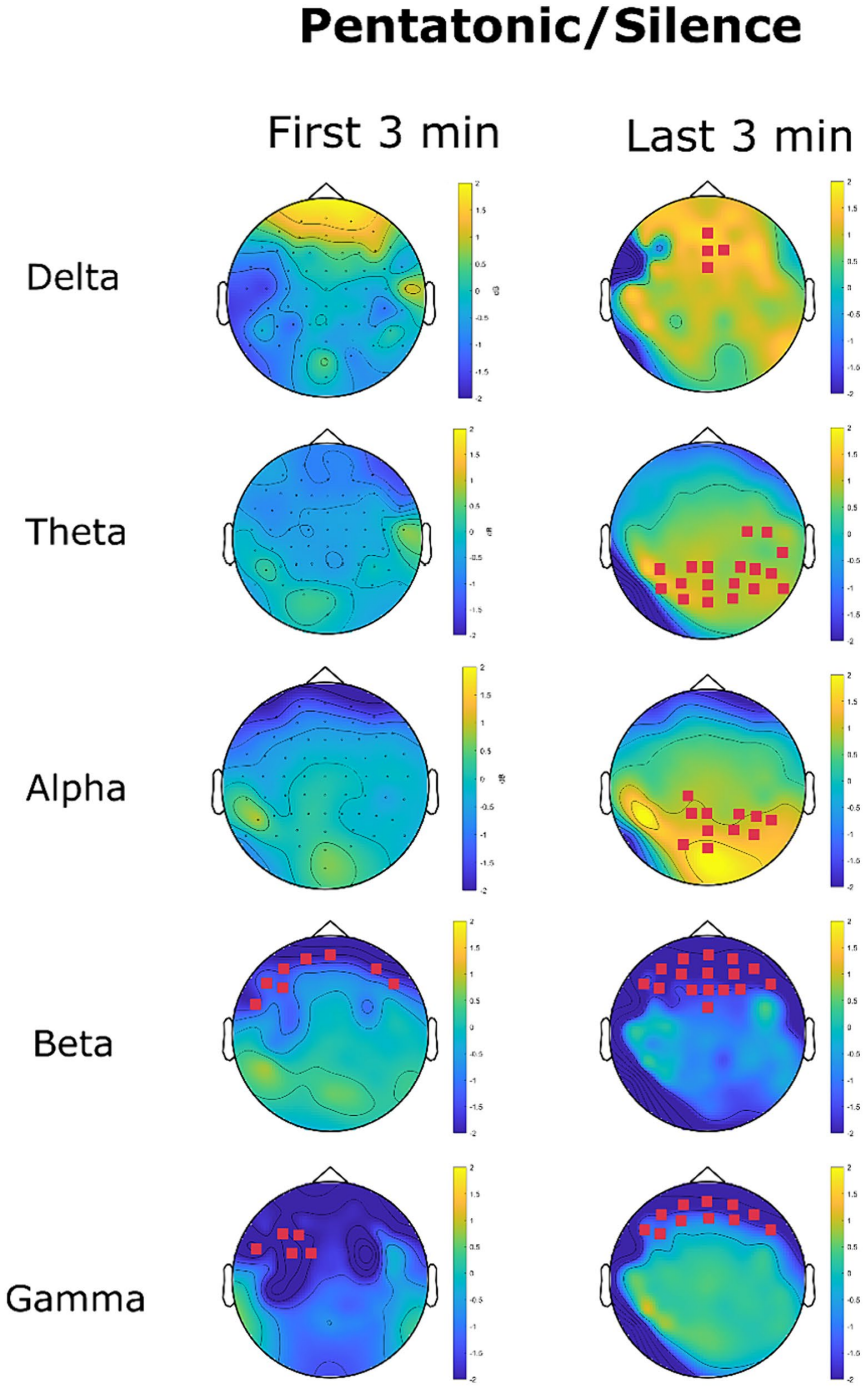
EEG power spectrum change (dB) in the pentatonic condition with reference to the silence condition. Magenta channel marks highlight the channel with a significant difference in power spectrum between the two conditions.

Figure 11 shows the changes in EEG oscillations in the pentatonic condition in comparison to the silence condition. A significant increase in delta oscillations was found in the last 3 min at frontal channels AFz, Fz, F2, and FCz. The mean power spectrum in the silence condition was 1.46 μV^2^/Hz (*SD* = 0.01), and 2.08 μV^2^/Hz (*SD* = 0.009) in the beats condition (*p* = 0.02, *d* = 62).

Theta oscillation recorded an increase in the last three minutes in parietal and parieto-occipital channels. In the last three minutes mean power spectrum density for theta oscillations was 0.22 μV^2^/Hz (*SD* = 0.22) in the silence condition and 2.08 μV^2^/Hz (*SD* = 0.09) in the pentatonic condition (*p* = 0.01, *d* = 11.06).

The same pattern was recorded for alpha oscillations, with a significant increase in 11 parietal, and parieto-occipital channels in the last three minutes. Mean power spectrum density increased from 0.05 μV^2^/Hz (*SD* = 0.44) in the silence condition to 0.07 μV^2^/Hz (*SD* = 0.64) in the pentatonic condition (*p* = 0.03, *d* = 0.03).

For all fast oscillations (beta and gamma) the pattern was a significant decrease in comparison to the silence condition. The decrease was limited to frontal sensors and persisted for the whole duration of the acoustic stimulation, being present both in the first and in the last three minutes.

For beta oscillations, the decrease in power spectrum in the first three minutes was from 0.11 μV^2^/Hz (*SD* = 0.03) in the silence condition to 0.07 μV^2^/Hz (*SD* = 0.31) in the pentatonic condition (*p* = 0.04, *d* = 0.18). The significant cluster was composed of six channels. In the last three minutes, beta oscillations decreased from 0.11 μV^2^/Hz (*SD* = 0.03) in the silence condition to 0.07 μV^2^/Hz (*SD* = 0.02) in the pentatonic condition (*p* = 0.02, *d* = 1.56). The significant cluster was composed of 11 frontal channels.

A similar pattern was recorded for gamma oscillations. In the first three minutes, the decrease affected five frontal sensors (F3, F5, FT7, FC3, FC1). The mean gamma power spectrum was 0.05 μV^2^/Hz (*SD* = 0.01) in the silence condition and 0.02 μV^2^/Hz (*SD* = 0.005) in the pentatonic condition (*p* = 0.05, *d* = 3.79). In the last three minutes, the decrease in gamma oscillations affected 16 frontal channels. The mean gamma power spectrum density was 0.05 μV^2^/Hz (*SD* = 0.02) in the silence condition and 0.03 μV^2^/Hz (*SD* = 0.009) in the pentatonic condition (*p* = 0.01, *d* = 1.28).

##### Comparison between the two acoustic conditions

No significant differences were found between beats and pentatonic sequence in all the EEG spectral bands, both in the first and last three min.

## Discussion

Auditory stimulations and music, in particular, have an important impact on arousal regulation (Bartlett, 1996; Schäfer et al., 2013). According to Schäfer et al. (2013), arousal and mood regulation are the most important dimensions of music listening. Unfortunately, the use of complex music for arousal regulation is strongly influenced by music preference (Jiang et al., 2013, 2016) with large individual differences. Finding relaxing music that is effective in a large population is therefore unviable. On the other end, auditory stimuli that consist of mere loudness modulation of pure tones (beats), are perceived as repetitive, unpleasant, and monotonous as highlighted by Watkins (2008), Recuero et al. (2013), and Engelbregt et al. (2021) and strongly confirmed also in our two studies. For these reasons, the use of beats to facilitate relaxation seems likewise problematic.

In our two studies we have tested an alternative auditory stimulation aimed to facilitate relaxation that is sufficiently complex and musical to be perceived as pleasant, and sufficiently simple and proto-musical to be appreciated by listeners with different musical backgrounds. The properties of the stimulus that can have contributed to the effect of inducing a relaxation response could be: (a) the very low oscillation created by the sequence of notes at 0.2 Hz (i.e., 5 s for each note); (b) the envelope with a slow attack and decay times that created a very smooth transition between notes, avoiding any percussive transition; (c) the use of a pentatonic scale that did not include semitones, being semitones responsible for the creation of a psychological tension toward the tonic, dominant, subdominant of the scale (Costa and Nese, 2020); (d) the use of a low-register with fundamentals being included in the octave ranging from C3 (130.81 Hz) to C4 (261.63 Hz). Low register sounds are perceived as more relaxing because a sense of alertness, distress, alarm is conveyed by an increase in pitch in both the vocal and musical domain (e.g., Rodero, 2011; Costa, 2013); (e) the use of a minor scale, being minor mode more associated with closure, intimacy, sadness than major mode (Parncutt, 2014); (f) the use of the simplest timbre possible: pure tones composed by only one sinusoidal function for the fundamental.

Many of these features (i.e., 0.2 Hz frequency, slow attach and release time, low register, pure-tone timbre) were shared also in monaural beats. The only parameters that differed between the two stimuli were the method for rendering the 0.2 Hz oscillation, the use of the pentatonic scale, and the use of a minor mode. In beats, the 0.2 Hz oscillation was created by the loudness amplitude modulation induced by the interference between two adjacent tones and in the pentatonic sequence the oscillation was rendered with the succession of notes. This is the first study that has investigated the EEG spectral perturbations induced by an oscillation created by a sequence of notes.

The frequency of 0.2 Hz in the second study was chosen as a direct consequence of the results of the first study that strongly evidenced an inverse linear function between frequency and relaxation ratings. Compared with 2 and 4 Hz, 0.2 Hz beats and pentatonic sequences were evaluated as more relaxing. The choice of such a low frequency (corresponding to a metronomic tempo of only 12 beats per minute) is of scientific interest because it is extraneous to the EEG delta range and to music practice, being considered too slow as a tempo for the creation of melodic lines that would be perceived with a sense of unity.

Most of the previous literature on the effects of beats on arousal, cognitive, and motor functions have used oscillations encompassed in the EEG range because the main explication factor was neural entrainment (Orozco Perez et al., 2020; Ingendoh et al., 2023). The effects of binaural beats are mainly attributed to their capacity to drive neural oscillations at the beat frequency (Pratt et al., 2010) but also effects of cross-frequency modulations have been reported. For example, Solcà et al. (2016) found that beat frequencies in the theta frequency range had an effect in increasing interhemispheric coherence at alpha frequency. In our study we found that 0.2 Hz oscillations failed to entrain brain activity on this specific frequency but cross-modulated delta, theta, and alpha with an increase in these specific bands, and beta and gamma, with a decrease of their spectrum power. An external oscillation could have effects not directly related to the frequency of the oscillation but also to EEG bands extraneous to the stimulating frequency.

A question that remains open is the selectivity of the 0.2 Hz frequency in explaining the effects of beats and pentatonic sequences found in this study. Previous studies that focused on the effects of binaural beats for the induction of relaxation or sleep have used a beat frequency on the delta and/or theta range (e.g., Padmanabhan et al., 2005; Dabiri et al., 2022). This is the first study that has used beats and acoustic sequences in the sub-delta range and future studies should ascertain if 0.2 Hz can be considered as a boundary or if the effect would be replicated also at lower frequencies.

Both beats and pentatonic sequences differed from silence in inducing a deeper relaxing state, as evidenced by the increase of the slow frequencies of the EEG spectrum. Specifically, in the beats condition there was an increase in theta oscillations that covered a whole area in the right temporal–parietal regions. Right hemispheric lateralization for theta oscillations in the beats condition could be associated with the unpleasant rating of this stimulus. Previous research has shown that unpleasant or dissonant chords as well as musical stimuli with negative valence show a right lateralization (Proverbio et al., 2016; Proverbio and Russo, 2022). In pentatonic sequences, the increase was not limited to theta oscillations but extended to delta and alpha oscillations. Furthermore, in the pentatonic sequence the hemispheric lateralization of theta waves largely disappeared in connection with the higher appreciation and pleasantness of these auditory stimuli in comparison to beats.

Theta waves are typically associated with relaxation, parasympathetic activation, and sympathetic withdrawal (e.g., Benson et al., 1981; McConnell et al., 2014). In the pentatonic condition, we also recorded augmented delta waves. An increase in delta waves through binaural beats has been demonstrated to have a significant effect in lowering anxiety (Le Scouarnec et al., 2001; Padmanabhan et al., 2005) and stress. In addition, Dabiri et al. (2022) showed that auditory stimulation with delta binaural beats enhanced sleep parameters reducing sleep onset, and awakenings during sleep. The increase in theta oscillations in both beats and pentatonic sequences and the increase in delta oscillations in pentatonic sequences were recorded only in the last three minutes of the 10-min stimulation, showing that the effect is not immediate and needs more than three minutes of listening to develop.

In the first three minutes, we recorded a strong reduction of beta/gamma spectral power in both beats and pentatonic sequences in comparison to the silence condition. Beta oscillations are typically associated with increased alertness. A decrease in beta activity has been correlated to a decrease in alertness (Gola et al., 2012), whereas an increase in beta and gamma power has been associated with cortical activation (Bekisz and Wróbel, 1999; Herculano-Houzel et al., 1999; Von Stein and Sarnthein, 2000). Gamma oscillations are enhanced in response to aversive or highly arousing stimuli compared to neutral pictures (Oya et al., 2002; Balconi and Lucchiari, 2008), and a connection between EEG gamma activity and emotion with special emphasis on negative emotional processing has been suggested by Matsumoto et al. (2006), Luo et al. (2007), and Oathes et al. (2008). Gamma has been shown to decrease during periods of relaxation and to increase during periods of imagining negative emotional material (Sebastiani et al., 2003). In Oathes et al. (2008) the authors showed that gamma oscillations were significantly increased in a worry task and significantly decreased in a relaxation induction period in comparison to a resting baseline period. In this perspective, the reduction of beta and gamma oscillations in the acoustic conditions could be associated with a reduction of cortical activation, a reduction of attentional processes, and an increased relaxation.

Since the effect of an increased relaxation was present in both the beats and the pentatonic sequence condition, we can conclude that this effect was largely due to the 0.2 Hz auditory oscillation and not to the specific method in which the oscillation was created. The specific method had significant effects on the subjective ratings, being pentatonic sequences rated significantly more pleasant than beats in both the first and second study. The fact that auditory stimulations had a higher impact on relaxation than silence confirms previous results found by Paszkiel et al. (2020) who investigated the impact of different sounds on stress levels, finding that relaxing music and music triggering an autonomous sensory meridian response were more effective in inducing calmness than silence. Furthermore, Schäfer et al. (2015) found that silence, as an acoustic scenario during the waking state, increased the experience of stress and danger in comparison to a musical acoustic scenario. The increased relaxation in the acoustical condition than in the silence condition in our study is probably explained considering that silence in a condition of no sleep is triggering the default mode network (Menon, 2023) that fosters daydreaming, mind-wandering, self-reflection, engaging the mind in active cognitive processes. Beats and pentatonic sequences could have contributed to the reduction of the default mode network, as was previously demonstrated in the case of meditation practices (Garrison et al., 2015).

The results of the study could be directly applied to implementing apps or devices that would facilitate relaxation and sleep induction. Pentatonic sequences at 0.2 Hz can be used as background acoustic stimulation whenever a reduction of arousal, an increase of relaxation, and the induction of a hypnagogic state is to be fostered. Future research could test if 0.2 Hz beats or pentatonic sequences could be applied also for the reduction of anxiety and the facilitation of sleep onset. On the musical level random pentatonic sequences were evaluated as more pleasant than beats, and for their properties of lacking semitones intervals and tonal tension they were particularly effective in creating a sensation of vagueness, and indetermination, that could facilitate relaxation when presented with low tempo.

## Data availability statement

The raw data supporting the conclusions of this article will be made available by the authors, without undue reservation.

## Ethics statement

The study was approved by the Bioethics Committee of the University of Bologna (Protocol # 0250471 10/17/2022). The studies were conducted in accordance with the local legislation and institutional requirements. The participants provided their written informed consent to participate in this study.

## Author contributions

MC: Conceptualization, Data curation, Formal analysis, Investigation, Methodology, Software, Supervision, Validation, Writing – original draft, Writing - review & editing. CV: Writing – original draft, Conceptualization, Data curation, Formal analysis, Investigation, Methodology, Software, Writing – review & editing. MO: Writing – review & editing, Conceptualization, Data curation, Investigation, Methodology. LT: Writing – review & editing, Conceptualization, Data curation, Investigation, Methodology. NP: Writing – review & editing, Conceptualization, Methodology. VN: Writing – review & editing, Conceptualization, Investigation, Methodology, Supervision.

## Supplementary material

Supplementary Audio 1 Beats.wav.

Supplementary Audio 2 Pentatonic.wav.
